# Supplementary material for: A Global Screen for Assembly State Changes of the Mitotic Proteome by SEC-SWATH-MS
Source: Cell Syst. 2020 Feb 26;10(2):133–155.e6. doi: 10.1016/j.cels.2020.01.001 (PMC7042714; doi:10.1016/j.cels.2020.01.001)

P62380 | TBPL1_HUMAN | TBPL1 TLF TLP TLP21 TRF2 TRP
Monomer MW [kDa]: 20.887 Monomer expected elution fraction: 54

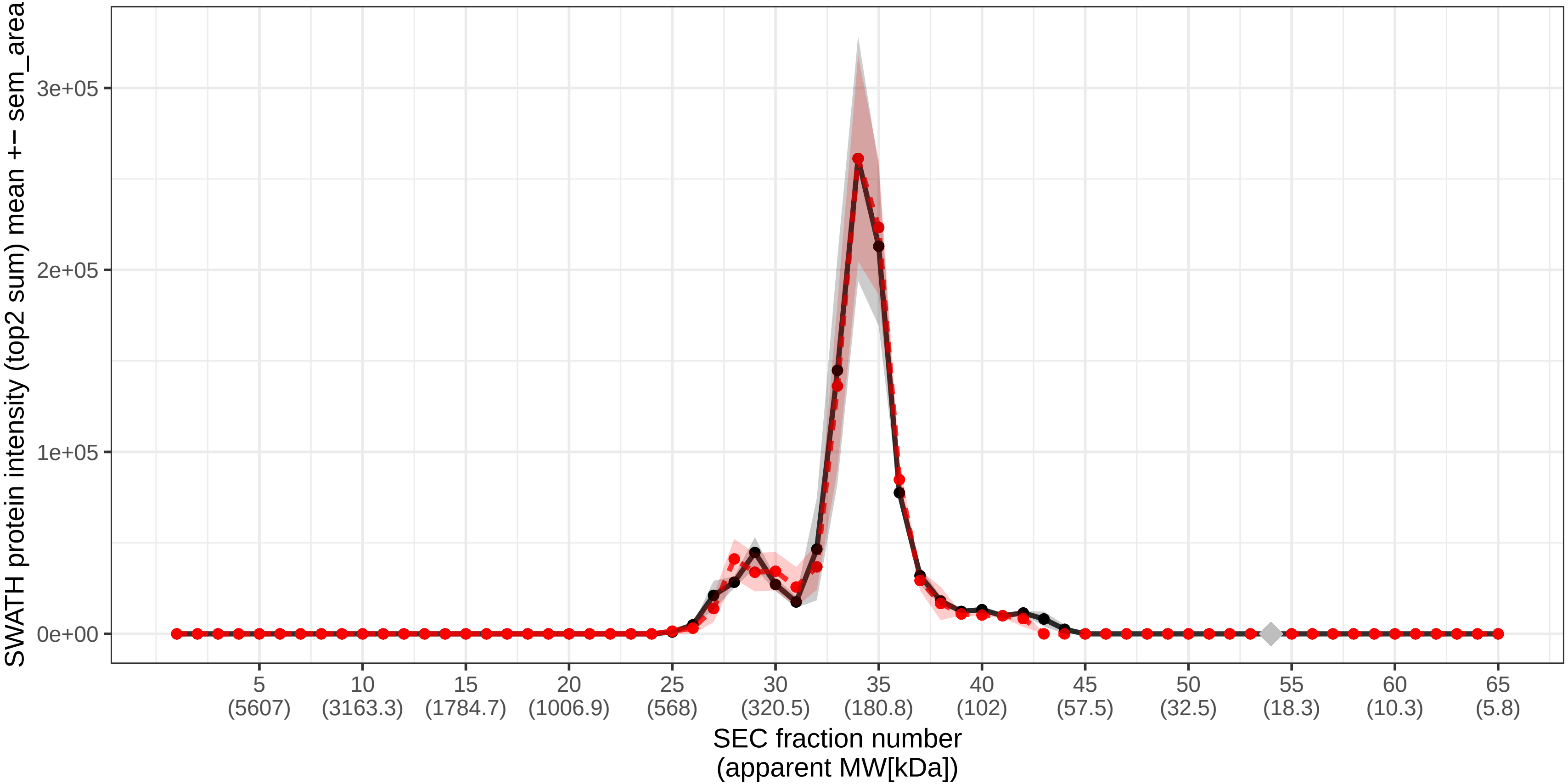

Supplement: Data S1. SEC-SWATH-MS Protein Chromatograms, Related to Figure 1 [file mmc6.zip › SECchrom_P62380_TBPL1_HUMAN_TBPL1_TLF_TLP_TLP21_TRF2_TRP.pdf]
